# Retraction: Regional impact of aging population on carbon dioxide emissions in China: Evidence from panel threshold regression (PTR)

**DOI:** 10.1371/journal.pone.0341200

**Published:** 2026-01-20

**Authors:** The PLOS One Editors

The *PLOS One* Editors retract this article [1] due to concerns about:

- Compromised peer review.
- High similarity to a previously published article [2] from the same author group that was not adequately discussed or acknowledged in [1] or declared at the time this article was submitted.
- Citation of multiple references that do not appear to support the corresponding cited statements, including References 3, 9, 11, 28, and 62.

The Editors have no evidence of any author involvement in the peer review concerns.

The Authors responded to acknowledge that Reference 62 (Lim et al. (1980)) was cited in error, and they stated that the other references were cited intentionally. The Editors do not consider this issue resolved.

YL, HX, BL, and LT did not agree with the retraction. NSM either did not respond directly or could not be reached.

## References

[1] Liang Y, Xinping H, Mazlan NS, Liang B, Ting L. RETRACTED: Regional impact of aging population on carbon dioxide emissions in China: Evidence from panel threshold regression (PTR). PLoS One. 2023;18(9):e0290582. doi: 10.1371/journal.pone.0290582 37708104 PMC10501635

[2] Liang Y, Mazlan NS, Mohamed AB, Mhd Bani NYB, Liang B. Regional impact of aging population on economic development in China: Evidence from panel threshold regression (PTR). PLoS One. 2023;18(3):e0282913. doi: 10.1371/journal.pone.0282913 36917591 PMC10013891

